# Molecular Characteristics of Extended-Spectrum Cephalosporin-Resistant Enterobacteriaceae from Humans in the Community

**DOI:** 10.1371/journal.pone.0129085

**Published:** 2015-06-01

**Authors:** Angela H. A. M. van Hoek, Leo Schouls, Marga G. van Santen, Alice Florijn, Sabine C. de Greeff, Engeline van Duijkeren

**Affiliations:** Centre for Infectious Disease Control (CIb), National Institute for Public Health and the Environment (RIVM), Bilthoven, The Netherlands; Institut National de la Recherche Agronomique, FRANCE

## Abstract

**Objective:**

To investigate the molecular characteristics of extended-spectrum cephalosporin (ESC)-resistant Enterobacteriaceae collected during a cross-sectional study examining the prevalence and risk factors for faecal carriage of extended-spectrum β-lactamase (ESBL)-producing Enterobacteriaceae in humans living in areas with high or low broiler density.

**Methods:**

ESC-resistant Enterobacteriaceae were identified by combination disc-diffusion test. ESBL/AmpC/carbapenemase genes were analysed using PCR and sequencing. For *E*. *coli*, phylogenetic groups and MLST were determined. Plasmids were characterized by transformation and PCR-based replicon typing. Subtyping of plasmids was done by plasmid multilocus sequence typing.

**Results:**

175 ESC-resistant Enterobacteriaceae were cultured from 165/1,033 individuals. The isolates were *Escherichia coli*(n=65), *Citrobacter freundii* (n=52), *Enterobacter cloacae* (n=38), *Morganella morganii* (n=5), *Enterobacter aerogenes* (n=4), *Klebsiella pneumoniae* (n=3), *Hafnia alvei* (n=2), *Shigella spp*. (n=2), *Citrobacter amalonaticus* (n=1), *Escherichia hermannii* (n=1), *Kluyvera cryocrescens* (n=1), and *Pantoea agglomerans* (n=1). The following ESBL genes were recovered in 55 isolates originating from 49 of 1,033 (4.7 %) persons: *bla*_CTX-M-1_ (n=17), *bla*_CTX-M-15_ (n=16), *bla*_CTX-M-14_ (n=9), *bla*_CTX-M-2_ (n=3), *bla*_CTX-M-3_ (n=2), *bla*_CTX-M-24_ (n=2), *bla*_CTX-M-27_ (n=1), *bla*_CTX-M-32_ (n=1), *bla*_SHV-12_ (n=2), *bla*_SHV-65_ (n=1) and *bla*_TEM-52_ (n=1). Plasmidic AmpC (pAmpC) genes were discovered in 6 out of 1,033 (0.6 %) persons. One person carried two different *E*. *coli* isolates, one with *bla*_CTX-M-1_ and the other with *bla*_CMY-2_ and therefore the prevalence of persons carrying Enterobacteriaceae harboring ESBL and/or pAmpC genes was 5.2 %. In eight *E*. *coli* isolates the AmpC phenotype was caused by mutations in the AmpC promoter region. No carbapenemase genes were identified. A large variety of *E*. *coli* genotypes was found, ST131 and ST10 being most common.

**Conclusions:**

ESBL/pAmpC genes resembled those from patients in Dutch hospitals, indicating that healthy humans form a reservoir for transmission of these determinants to vulnerable people. The role of poultry in the transmission to humans in the community remains to be elucidated.

## Introduction

Extended-spectrum-β-lactamase/AmpC producing Enterobacteriaceae have been found among humans worldwide. Most large-scale studies in humans, however, report data of patients or travelers and/or focus on ESBL-producing bacteria and/or certain bacterial species only (e.g. *Escherichia coli* or *Klebsiella pneumoniae*) [1–4]. Consequently, data on the prevalence of fecal carriage of ESBL/AmpC/carbapenemase producing Enterobacteriaceae in healthy humans in the community are scarce. The major mechanism of resistance to extended spectrum cephalosporins (ESC) in the family Enterobacteriaceae is the production of an extended-spectrum β-lactamase (ESBL) or an AmpC β-lactamase [5]. ESBLs are often plasmid mediated, while the production of AmpC β-lactamases can result either from (over)expression of the chromosomal *ampC* gene or by the acquisition of a plasmid-mediated *ampC* determinant [5]. Initially ESBL/AmpC-producing organisms were associated with hospitals and institutional care in humans, but they are now increasingly found in the community and in food-producing animals [6]. A connection between ESBL/AmpC-producing bacteria in food animals and humans has been suggested [1, 7–9]. ESBL/AmpC-producing Enterobacteriaceae have frequently been reported in broilers and therefore they have been considered as a reservoir for ESBL/AmpC-encoding resistance genes [7, 10]. Transmission from broilers to humans through the food chain has been proposed [11–13], but could also occur through direct contact or through the environment [7]. In 2011, a cross-sectional study was performed to determine the prevalence of, and identify risk factors for, carriage of ESBL-producing Enterobacteriaceae in people living in municipalities with either high or low broiler densities [14]. The prevalence of carriage of ESBL-producing bacteria was 5.1% and this percentage was lower in municipalities with high broiler densities (3.6%) compared to municipalities with low broiler densities (6.7%) [14]. The aim of the present study was to analyse the isolates from this cross-sectional study, including isolates with an AmpC phenotype, with respect to molecular characteristics and compare them to published data on isolates from patients, broilers, and persons in contact with broilers.

## Materials and Methods

A cross-sectional study was conducted between August and December 2011. A random sample of adults (>18 years), stratified according to age and gender was taken from eight municipalities across 4 provinces of the Netherlands: North-Brabant, Gelderland, Overijssel and Frisia. In each province the municipality with the highest respectively lowest number of broiler farms per km^2^ was selected. This information was obtained from the Dutch Product Board for Poultry and Eggs. In total, 3,949 individuals were contacted by post and were asked to return a rectal swab and a questionnaire on demographics, contact with animals, lifestyle, medical history, eating habits and travel. For each respondent, distance to the nearest broiler farm was obtained using geographic data. Exclusion criteria were living or working on a commercial broiler farm [14]. The study was approved by the Medical Ethics Committee of University Medical Centre Utrecht, The Netherlands (protocol number 11–277). All participants provided written informed consent. Rectal swabs were obtained from 1,033 persons and were analysed to determine the presence of ESBL/AmpC/carbapenemase-producing Enterobacteriaceae. Bacteria were isolated by selective enrichment (Luria-Bertani broth (MP Biomedicals, Amsterdam, the Netherlands) supplemented with 1mg/L cefotaxime (Sigma-Aldrich, Zwijndrecht, the Netherlands), and cultured on selective plates (MacConkey agar no. 3, Oxoid, Badhoevedorp, the Netherlands) supplemented with 1 mg/L cefotaxime). Isolates (1–6 per person, depending on the numbers of different phenotypes) were tested phenotypically for ESBL/AmpC-production by a combination disc-diffusion test using cefotaxime and ceftazidime discs, with and without clavulanic acid (Becton Dickinson B.V., Breda, the Netherlands), according to CLSI guidelines [15]. A cefoxitin disc (Becton Dickinson B.V., Breda, the Netherlands) was used to detect AmpC phenotypes [7]. Genotypes of the ESBL/AmpC-positive isolates were determined by PCR and gene sequencing. For isolates with an ESBL phenotype, primers detecting CTX-M-group 1, CTX-M-group 2, CTX-M-group 9, CTX-M-group 8/25, OXA-1 like, SHV and TEM were used. In case of isolates displaying an AmpC phenotype, primers specific for ACC, ACT, BIL, CMY, DHA, FOX, LAT, MIR and MOX were used. In addition, all 71 isolates with an ESBL-phenotype were investigated using primers for the detection of carbapenemase genes of the KPC, NDM, OXA-48, and VIM families. For *E*. *coli* isolates with an AmpC phenotype, but negative in PCR for β-lactamase genes, chromosomal *ampC* promoter mutations were detected by PCR and sequencing analysis (Table 1).

**Table 1:** Primers used to completely sequence the ESBL/AmpC resistance genes.

Gene family	Primer (5’-3’)	Purpose	Reference
ACC group	gcatgctgattggcgtgc	PCR & Sequencing	This study
	cagccgctgatgcagaag	Sequencing	
	ccccatattggcttgcac	Sequencing	
	agggcgtgctgtaatacc	PCR & Sequencing	
ACT & MIR group	cacagtcaaatccaacagac	PCR & Sequencing	This study
	ctataagtaaaaccttcaccg	Sequencing	
	cgtaatgcgcctcttccg	Sequencing	
	tttttgtaggccgggtaag	PCR & Sequencing	
	agcgccacccggcaatg	PCR & Sequencing	
AmpC promoter region	aatgggttttctacggtctg	PCR & Sequencing	[16]
	gggcagcaaatgtggagcaa	PCR & Sequencing	
CMY-2 group	atgatgaaaaaatcgttatgctgc	PCR & Sequencing	[17]
	ctccagcattggtctgtttg	Sequencing	This study
	agttcagcatctcccagcc	Sequencing	
	gcttttcaagaatgcgccagg	PCR & Sequencing	[18]
CTX-M-1 group	gtgtgagaagcagtctaaa	PCR & Sequencing	This study
	cggaaggagaaccaggaa	PCR & Sequencing	
	Ctgggtgtggcattgatt	Sequencing	
	Ctgggtaaagcattgggt	Sequencing	
	Cccgaggtgaagtggtat	Sequencing	
	Gcacacttcctaacaaca	Sequencing	
CTX-M-2 group	Atgatgactcagagcattcg	PCR & Sequencing	[19]
	Ttattgcatcagaaaccgtg	PCR & Sequencing	
CTX-M-9 group	Tggtgacaaagagagtgcaacg	PCR & Sequencing	[20]
	Tcacagcccttcggcgat	PCR & Sequencing	
DHA group	Gtgaatctgacgatacttgc	PCR & Sequencing	This study
	Tcacaggtgtgctgggtg	Sequencing	
	Taaccgtacgcatactggc	Sequencing	
	Aataatcttaaattacggccc	PCR & Sequencing	
	Tccgcaggggcctgttcag	PCR & Sequencing	
SHV	Ttatctccctgttagccacc	PCR & Sequencing	[17]
	Gatttgctgatttcgctcgg	PCR & Sequencing	
TEM	Gcggaacccctatttg	PCR & Sequencing	[21]
	Accaatgcttaatcagtgag	PCR & Sequencing	

DNA was extracted by Chelex-100 chelating resin (Bio-Rad Laboraties B.V., Veenendaal, the Netherlands). Published primer sets were used to screen for the group of ESBL/AmpC gene [22]. Complete ESBL/AmpC gene sequences were obtained by PCR using the primers as indicated in Table 1. Resulting amplicons were treated with ExoSAP-IT (Isogen Life Science, De Meern, the Netherlands) according to manufacturers’ instructions. Aliquots of the purified PCR products were used in sequence reactions on an AB 3730 genetic analyser using the Big Dye Terminator technology (Applied Biosystems, Bleiswijk, the Netherlands). Each sequence was compared with known β-lactamase gene sequences (www.lahey.org/Studies) by multiple-sequence alignment using the BLAST, BioNumerics and Seaview programmes.

Phylogenetic groups were determined for *E*. *coli* according to Doumith *et al*. [23]. Strains were sub-grouped according to Escobar-Páramo *et al*. [24]. For isolates identified as non-*E*. *coli* the bacterial species was identified by BBL (Becton Dickinson B.V., Breda, the Netherlands) and MALDI TOF MS on a Bruker Microflex LT instrument (Bruker Daltonics GmbH, Bremen, Germany).

Multilocus sequence typing (MLST) of *E*. *coli* was performed according to Wirth *et al*. [25]. Plasmids were characterised on a selection of isolates representing different ESBL/AmpC-genes and phylogenetic groups. Plasmids were first isolated using QIAfilter Plasmid Midi Kit (QIAGEN Benelux B.V., Venlo, the Netherlands). Next, the isolated plasmids were transformed into ElectroMAX DH10B cells (Invitrogen, Bleiswijk, the Netherlands) by electroporation [26]. The resulting transformants were cultured on selective plates (LB agar (MP Biomedicals, Amsterdam, the Netherlands) supplemented with 1 μg/ml cefotaxime) to isolate recipients carrying an ESBL/AmpC plasmids. PCR-based replicon typing (PBRT) was conducted to classify the plasmid inside the transformant using the PBRT kit (Diatheva, Fano, Italy), according to Carattoli *et al*. [27]. IncF, IncI1 and IncN plasmids were further characterized by plasmid MLST (pMLST) [28, 29, 30].

## Results

Out of 1,033 persons investigated, 165 (15.9%) carried ESC-resistant Enterobacteriaceae. Ten persons were positive for two types of ESC-resistant Enterobacteriaceae, yielding a total of 175 isolates with an ESBL/AmpC resistance phenotype.

### Species identification

Species identification of the 175 isolates showed that they were *Escherichia coli* (n = 65), *Citrobacter freundii* (n = 52), *Enterobacter cloacae* (n = 38), *Morganella morganii* (n = 5), *Enterobacter aerogenes* (n = 4), *Klebsiella pneumoniae* (n = 3), *Hafnia alvei* (n = 2), *Shigella spp*. (n = 2), *Citrobacter amalonaticus* (n = 1), *Escherichia hermannii* (n = 1), *Kluyvera cryocrescens* (n = 1), and *Pantoea agglomerans* (n = 1).

### ESBL/AmpC phenotype and genes of all isolates

Of these 175 isolates, 119 (68.0%) showed an AmpC-phenotype and were recovered from 116 persons. For most isolates, however, no AmpC gene was found. If an AmpC gene was detected that is specific for the species concerned (e.g. *bla*_CMY-2_ in *C*. *freundii*, *bla*_ACC_ in *H*. *alvei*, *bla*_DHA_ in *M*. *morganii*, and *bla*_ACT/MIR-1_ in *Enterobacter* species) it was considered as chromosomal and these isolates were excluded from further analysis. Six isolates carried plasmidic AmpC (pAmpC) genes: 4 *E*. *coli* isolates, 1 *P*. *agglomerans* isolate and 1 *C*. *freundii* isolate. The prevalence of pAmpC-producing Enterobacteriaceae was 5.0% (6/119) of the isolates with an AmpC phenotype. The prevalence of persons carrying a pAmpC-positive isolate was 0.6% (6/1,033). The remaining 56 (32.0%) ESC-resistant isolates displayed an ESBL-phenotype. In 55 (98.2%) of these 56 isolates an ESBL-gene was found. Isolates carrying an ESBL-gene were *E*. *coli* (n = 51), *E*. *hermannii* (n = 1), *Klebsiella pneumonia* (n = 2) and *Citrobacter freundii* (n = 1) and were recovered from 49 persons yielding an ESBL prevalence of 4.7% (49/1,033). The most frequently identified ones were *bla*_CTX-M-1_ (n = 17), *bla*_CTX-M-15_ (n = 16) and *bla*_CTX-M-14_ (n = 9). For one *E*. *coli* isolate no ESBL gene was found, but the isolate carried *bla*_OXA-1_ and *bla*_TEM-1b_.

Three persons carried two isolates with different ESBL-genes: two of them (P33, P39) carried two different *E*. *coli* genotypes with *bla*_CTX-M-14_ and *bla*_CTX-M-15,_ respectively, and one individual (P1) carried a *C*. *freundii* with *bla*_CTX-M-15_ and an *E*. *coli* with *bla*_CTX-M-3_. Two persons (P17, P44) carried two *E*. *coli* with different MLSTs with the same ESBL-gene. One individual (P14) carried two *E*. *coli* isolates with *bla*_CTX-M-1_, but one of these isolate also contained *bla*_TEM-1b_. One person (P3) carried an *E*. *coli* with *bla*_CTX-M-1_ and an *E*. *coli* carrying *bla*_CMY-2_. The prevalence of persons carrying an ESBL/pAmpC positive isolate was 5.2 (54/1,033).

No genes encoding for the production of carbapenemases were found.

### Characteristics of the *E*. *coli* isolates

AmpC genes were found in only four of the 13 phenotypically AmpC *E*. *coli* isolates (*bla*_CMY-2_ (n = 3) and *bla*_DHA-1_ (n = 1)). However, eight of the isolates belonging to MLST ST88, ST95, ST131 (n = 2), ST345, ST453 (n = 2) and ST500 carried mutations in the promoter region of *ampC* (−42T−18A−1T+58T+81G (n = 4); −32A−28A (n = 2); −32A−28A+17T+30A (n = 1) and −18A−14INS(G)−1T+58T+81G (n = 1). Most of the 65 *E*. *coli* isolates displayed an ESBL-phenotype (n = 52). The predominant ESBL-genes in *E*. *coli* were *bla*_CTX-M-1_ (n = 17), *bla*_CTX-M-15_ (n = 13) and *bla*_CTX-M-14_ (n = 9). Other ESBL-genes found were *bla*_CTX-M-2_ (n = 3), *bla*_CTX-M-3_ (n = 2), *bla*_CTX-M-24_ (n = 2), *bla*_SHV-12_ (n = 2), *bla*_CTX-M-27_ (n = 1), *bla*_CTX-M-32_ (n = 1), *bla*_TEM-52_ (n = 1). Other β-lactamase genes found were *bla*_TEM-1b_ (n = 20), *bla*_TEM-84_ (n = 1), and *bla*_OXA-1_ (n = 6) (Table 2).

**Table 2:** Characteristics of the ESBL/pAmpC-producing isolates.

Person	Bacterial species	Isolate^a^	Phylogroup	MLST	ESBL/AmpC gene	Other β-lactamase gene	Plasmid	pMLST or FAB formula
P1	*C*. *freundii*	0754_1			CTX-M-15		nd	
	*E*. *coli*	0754_6	A1	ST10	CTX-M-3		nd	
P2	*C*. *freundii*	2090_2			DHA-1		nd	
P3	*E*. *coli*	0331_3	B2	ST131	CMY-2		incI1	ST12 (CC-12)
	*E*. *coli*	0331_4	D1	ST69	CTX-M-1		incI1	ST36 (CC-5)
P4	*E*. *coli*	3745_1	A0	ST93	CMY-2		incA/C	
P5	*E*. *coli*	1517_1	B2_2_	ST219	CMY-2		incI1	ST12 (CC-12)
P6	*E*. *coli*	3325_1	A1	ST10	CTX-M-1	TEM-1b	nd	
P7	*E*. *coli*	3554_3	A1	ST10	CTX-M-1	TEM-1b	incI1	ST58 (CC-58)
P8	*E*. *coli*	3841_1	D1	ST59	CTX-M-1		incI1	ST58 (CC-58)
P9	*E*. *coli*	1349_4	A1	ST88	CTX-M-1		nd	
P10	*E*. *coli*	2079_1	B1	ST58	CTX-M-1	TEM-1b	NTP	
P11	*E*. *coli*	2115_1	B1	ST58	CTX-M-1	TEM-1b	incI1	ST7 (CC-7)
P12	*E*. *coli*	2326_1	A1	ST10	CTX-M-1	TEM-1b	nd	
P13	*E*. *coli*	2555_5	B1	ST58	CTX-M-1	TEM-1b	incI1	ST58 (CC-58)
P14	*E*. *coli*	2643_1	B1	ST5037	CTX-M-1		incN	ST1
	*E*. *coli*	2643_5	B1	ST5037	CTX-M-1	TEM-1b	nd	
P15	*E*. *coli*	2668_1	A1	ST88	CTX-M-1	TEM-1b	incI1	ST3 (CC-3)
P16	*E*. *coli*	2760_1	B1	ST2536	CTX-M-1		incI2	
P17	*E*. *coli*	2865_3	D2	ST657	CTX-M-1		incN	ST1
	*E*. *coli*	2865_5	A1	ST744	CTX-M-1	TEM-1b	nd	
P18	*E*. *coli*	2870_1	D1	ST59	CTX-M-1		incI1	ST58 (CC-58)
P19	*E*. *coli*	2884_1	B1	ST5037	CTX-M-1		incN	ST1
P20	*E*. *coli*	0610_4	A1	ST10	CTX-M-2	TEM-1b	incF	F2:A−:B1
P21	*E*. *coli*	2632_1	D2	ST5038	CTX-M-2		nd	
P22	*E*. *coli*	2646_1	B1	ST1049	CTX-M-2		incY	
P23	*E*. *coli*	2316_3	A0	ST1178	CTX-M-3		nd	
P24	*E*. *coli*	0002_1	A1	ST10	CTX-M-14		NTP	
P25	*E*. *coli*	0164_2	B1	ST58	CTX-M-14		incI1	ST80
P26	*E*. *coli*	0413_1	D1	ST69	CTX-M-14		incF	F35:A−:B−
P27	*E*. *coli*	0482_3	D2	ST38	CTX-M-14	TEM-1b	nd	
P28	*E*. *coli*	2968_2	B2_3_	ST1982	CTX-M-14		incB/O	
P29	*E*. *coli*	3055_1	A1	ST5039	CTX-M-14		incI1	ST80
P30	*E*. *coli*	2921_2	D1	ST414	CTX-M-14		nd	
P31	*E*. *coli*	0247_1	D1	ST5041	CTX-M-15	TEM-1b	incI1	ST37
P32	*E*. *coli*	0391_3	D2	ST38	CTX-M-15		nd	
P33	*E*. *coli*	0782_2	B2_3_	ST131	CTX-M-15	OXA-1	incF	F2:A1:B−
	*E*. *coli*	0782_3	D2	ST648	CTX-M-14		incN	ST1
P34	*E*. *coli*	0915_2	D2	ST405	CTX-M-15	OXA-1	incF	F1:A1:B16
P35	*E*. *coli*	3681_1	A0	ST1314	CTX-M-15	OXA-1, TEM-1b	incK	
P36	*E*. *coli*	1899_3	B1	ST1664	CTX-M-15	TEM-1b	nd	
P37	*E*. *coli*	1396_1	D2	ST648	CTX-M-15		nd	
P38	*E*. *coli*	1405_3	D2	ST648	CTX-M-15	OXA-1, TEM-84	nd	
P39	*E*. *coli*	2102_1	A1	ST48	CTX-M-15	TEM-1b	nd	
	*E*. *coli*	2102_4	A1	ST746	CTX-M-14^b^	TEM-1b	incF	F77:A−:B−
P40	*E*. *coli*	2187_1	D2	ST38	CTX-M-15	TEM-1b	nd	
P41	*E*. *coli*	2622_1	A1	ST617	CTX-M-15	OXA-1	incF	F31:A4:B1
P42	*E*. *coli*	2767_1	B1	ST5040	CTX-M-15		incF	F78:A−:B47
P43	*E*. *coli*	2918_1	B1	ST5036	CTX-M-15		NTP	
P44	*E*. *coli*	2830_1	B1	ST58	CTX-M-24		incF	F35:A−:B−
	*E*. *coli*	2830_5	D2	ST38	CTX-M-24	TEM-1b	nd	
P45	*E*. *coli*	2108_3	B2_3_	ST131	CTX-M-27		incF	F1:A2:B20
P46	*E*. *coli*	2680_1	A0	ST540	CTX-M-32		incF	F−:A−:B38
P47	*E*. *coli*	1680_2	B2_3_	ST131	DHA-1		incF	F1:A1:B1
P48	*E*. *coli*	2256_1	B2_2_	ST12	SHV-12	TEM-1b	incI1	ST95
P49	*E*. *coli*	1002_1	A1	ST3877	SHV-12		NTP	
P50	*E*. *coli*	1480_2	D2	ST1163	TEM-52 ^c^		NTP	
P51	*E*. *hermannii*	2829_3			CTX-M-15		nd	
P52	*K*. *pneumoniae*	3199_3			CTX-M-15,		nd	
					SHV-11			
P53	*K*. *pneumoniae*	1241_3			SHV-65		nd	
P54	*P*. *agglomerans*	0952_1			CMY-48		nd	

^a^ The first four numbers of the isolate name indicate the person sampled, the number after the underscore indicates the isolate number.

^b^ The CTX-M-14 has four synonymous mutations (A372G, G570A, G702A, A875G) in comparison to the official entry for this allele (accession number AF252622).

^c^ The TEM-52 gene has one synonymous mutation (C228T) in comparison to the official entry for this TEM allele (accession number Y13612).

CC = clonal complex; nd = not determined; NTP = non-typeable incompatibility group plasmid.

The predominant *E*. *coli* phylogenetic groups found were B1 (n = 17) and A1 (n = 16), followed by D2 (n = 11), B2_3_ (n = 8), D1 (n = 6), A0 (n = 5), and B2_2_ (n = 2). The most prevalent *E*. *coli* MLST types were ST10 (n = 6), ST131 (n = 6), followed by ST58 (n = 5), and ST38 (n = 4) but a great diversity of different genotypes was found. Plasmid family incI1 was most commonly identified, followed by incF. pMLST revealed that within incI1 subtype ST58 was found most often. All incN plasmids had subtype ST1. For the incF plasmid family a variety of subtypes were found (Table 2).

### Characteristics of the non-*E*.*coli* isolates

The *E*. *hermannii* isolate carried *bla*_CTX-M-15_ and *bla*_OXA-1_. Of the *K*. *pneumoniae* isolates, two had an ESBL phenotype (Table 2); one harboured *bla*_SHV-65_, while the other contained *bla*_CTX-M-15_ and *bla*_SHV-11_, both genes had synonymous mutations. The third *K*. *pneumoniae* isolate displayed an AmpC phenotype, but no gene could be characterized. The *P*. *agglomerans* isolate carried *bla*_CMY-48_. The *C*. *freundii* isolate with the ESBL-phenotype contained *bla*_CTX-M-15_.

### Analysis of risk factors

After analysis of the questionnaires we found no clear evidence that certain genes were more often found in specific exposure categories. However, *bla*_CTX-M-1_ genes were relatively more often found than other genes among persons owning or in contact with a horse compared to persons not frequently exposed to a horse (p = 0,04) (Table 3).

**Table 3:** Distribution of ESBL/pAmpC-genes over the different risk categories.

		Number of persons carrying an isolate with *bla*_CTX-M-1_	Number of persons carrying an isolate with *bla*_CTX-M-15_	Number of persons carrying an isolate with other gene	Total number of positive persons
**Broiler density**	Low	11 (32.4%)	9 (26.5%)	14 (41.2%)	34
	High	4 (20.0%)	7 (35.0%)	9 (45.0%)	20
**Owning/contact with a pet**	No	4 (19.0%)	7 (33.3%)	10 (47.6%)	21
	Yes	11 (33.3%)	9 (27.3%)	13 (39.4%)	33
**Owning/contact with a horse**	No	10 (22.2%)	13 (28.9%)	22 (48.9%)	45
	Yes	5 (55.6%)	3 (33.3%)	1 (11.1%)	9
**Urinary tract infection**	No	12 (24.5%)	16 (32.7%)	21 (42.9%)	49
	Yes	3 (60.0%)	0 (0.0%)	2 (40.0%)	5
**Hospital admission**	No	6 (25.0%)	5 (20.8%)	13 (54.2%)	24
	Yes	9 (30.0%)	11 (36.7%)	10 (33.3%)	30
**Eating meat**	No	0 (0.0%)	0 (0.0%)	0 (0.0%)	0
	Yes	15 (27.8%)	16 (29.6%)	23 (42.6%)	54
**Travelling abroad**	No	8 (40.0%)	4 (20.0%)	8 (40.0%)	20
	Yes	7 (20.6%)	12 (35.3%)	15 (44.1%)	34

## Discussion

In the present study the prevalence of persons carrying Enterobacteriaceae harboring ESBL and/or pAmpC genes was 5.2%. Response analysis with respect to age, sex, and province and broiler density showed that a representative sample of Dutch adults was obtained [14]. Medical histories of 1,025/1,033 persons were available and 7.9% reported admission to a hospital and 6.4% urinary tract infection in the 6 month prior to inclusion in the present study and neither of these two factors was identified as a risk factor for being ESBL-positive [14]. Therefore, this study population represents a predominantly healthy general population. Most studies on ESBL/AmpC producing bacteria include either hospitalized patients or persons visiting a general practitioner.

To date only limited data are available on the prevalence of pAmpC-producing bacteria in the open population. The prevalence of persons carrying pAmpC genes in the present study (0.6%) was slightly lower than the 1.3% found in a study in community-dwelling individuals in the densely populated region of Amsterdam [31]. The disparity might be caused by the different study population: participants of the current study were living in rural areas, but dissimilarities in the methodology might also be an explanation. The prevalence of ESBL and pAmpC carriers in patients and out-patients of a Spanish University teaching hospital was 5.0% and 0.6%, respectively, which is similar to our findings [32]. The pAmpC gene discovered most frequently was *bla*_CMY-2_ [31, 32] and this corresponds with the findings of the present study. Altogether, the findings indicate that healthy humans form a reservoir for transmission of these determinants to vulnerable people.

Interestingly, only two persons carrying ESBL-positive *K*. *pneumoniae* were found (0.2% of all persons tested), although the prevalence of ESC-resistant *K*. *pneumoniae* is increasing in Dutch hospitals. The European Antimicrobial Resistance Surveillance System that collects resistance data from invasive isolates throughout Europe showed that third-generation cephalosporin resistance in The Netherlands has increased from 3.5% in 2005 to 7.5% in 2013 in *K*. *pneumoniae* [33]. This might be explained by the fact that ESBL-producing *K*. *pneumoniae* have different transmission dynamics compared to ESBL-producing *E*. *coli*, the predominant ESBL-positive species in the present study. A recent study showed a higher rate of community acquisition among ESBL-producing *E*. *coli* compared to ESBL-producing *K*. *pneumoniae* in patients with bacteremia [34]. In addition, ESBL-producing *E*. *coli* isolates had more different genotypes and patients infected with ESBL-producing *E*. *coli* were more likely to come from high prevalence countries compared to ESBL-producing *K*. *pneumoniae* supporting the notion that ESBL-producing *E*. *coli* is more likely to be acquired in community settings while *K*. *pneumoniae* is more often associated with hospital outbreaks and clonal transmission within the hospital [34].

One third of all ESC-resistant isolates in the present study carried an ESBL-gene and in all but one isolate with an ESBL-phenotype an ESBL-gene was found. *bla*_CTX-M-1_ was found most frequently and exclusively in *E*. *coli*, followed by *bla*_CTX-M-15_ and *bla*_CTX-M-14_. In a study investigating ESBL-producing Enterobacteriaceae in Dutch community patients with gastrointestinal complaints, the most prevalent ESBL gene was *bla*_CTX-M-15_, comprising 47% of all ESBL-genes and 85% of the genes of the CTX-M-group 1 [3]. In another study, *bla*_CTX-M-15_ was also most prevalent (39%), followed by *bla*_CTX-M-1_ (15%), among clinical Enterobacteriaceae obtained from Dutch patients [35]. Another Dutch study, however, found *bla*_CTX-M-1_ most often in faecal samples from persons admitted to the hospital, whereas *bla*_CTX-M-14_ was predominant in isolates from blood cultures, followed by *bla*_CTX-M-1_ [1]. In a German study investigating ESBL-producing *E*. *coli* in persons from the general population that had been in contact with patients with gastroenteritis, the majority of isolates belonged to CTX-M-type ESBL, with *bla*_CTX-M-15_ (46%) and *bla*_CTX-M-1_ (24%) as the most common types [4]. Therefore, in clinical isolates and persons in contact with patients, *bla*_CTX-M-15_ seems to be more prevalent than *bla*_CTX-M-1_ although both genes were found equally often in predominantly healthy persons in the community. Further research into the reason for this difference is needed. *bla*_CTX-M-1_ was most often associated with plasmids of the IncI1 family, while *bla*_CTX-M-15_ was more often associated with plasmids of the IncF family, underlining the different transmission dynamics.

Only few studies analyzed possible risk factors for carriage of ESBL/pAmpC-producers. Our results should be interpreted with caution, because of the small number of cases. Still, *bla*_CTX-M-1_ genes were relatively more often found among persons owning or in frequent contact with a horse. Interestingly, in a previous study contact with horses was identified as a risk factor for being ESBL-positive [14].

In this study, *E*. *coli* ST131, ST10, ST58 and ST38 were found most often. This is in accordance with the findings of Reuland *et al*. [3]: the predominant *E*. *coli* STs in their study were ST38, ST131, ST648 and ST10. *E*. *coli* ST131 has emerged as a global epidemic, multidrug-resistant clade. *E*. *coli* ST131 may cause extraintestinal infections, especially of the urinary tract, and its ESBL production is most often due to the presence of *bla*_CTX-M-15_ [36]. In an international study investigating 240 ESBL-producing *E*. *coli* with ST131 from nine countries, 193 (80%) contained *bla*_CTX-M-15_ [36]. In a Dutch study investigating clinical isolates most of the ST131 *E*. *coli* contained *bla*_CTX-M-15_, and presence of this gene was associated with higher levels of resistance [35]. In the present study only one person carrying *E*. *coli* ST131 containing *bla*_CTX-M-15_ was found indicating that this ST131 clade does not seem to be endemic in humans in the community in the Netherlands.

Most *E*. *coli* isolates belonged to phylogroups A1 and B1. These phylogroups are less often recovered from extraintestinal body sites. Isolates belonging to phylogroups B2 and D, however, were also found. Virulent strains causing extraintestinal infections belong mainly to groups B2 and D [24, 37]. This indicates that humans in the community carry *E*. *coli* isolates that have the potential to cause disease.

The prevalence of ESBL carriage was higher in areas with low broiler densities than in areas with high broiler densities and therefore living in areas with high broiler density was not identified as a risk factor [14]. The prevalence of ESBL/pAmpC carriage among people on broiler farms (19.1%) was higher than in the present study and an increased risk of carriage was shown among individuals having a high degree of contact with live broilers [7]. The most prevalent ESBL/AmpC genes in isolates from humans on broiler farms as well as broilers were *bla*_CMY-2_, *bla*_CTX-M-1_ and *bla*_SHV-12_, followed by *bla*_TEM-52_, while *bla*_CTX-M-15_ was not found [7, 10]. In contrast, in the present study, *bla*_CTX-M-1_, *bla*_CTX-M-15_ and *bla*_CTX-M-14_ were among the most prevalent ESBL-genes identified and *E*. *coli* isolates carrying *bla*_CMY-2_, *bla*_SHV-12_ and *bla*_TEM-52_ were only found sporadically although 94.5% of the study participants reported eating chicken meat [14]. It has been postulated that humans acquire ESBL-producing bacteria by eating chicken meat, because Dutch chicken meat has been shown to be contaminated with *E*. *coli* strains containing ESBL-genes similar to those found in patients [1, 12]. The same genes are, however, present in many different potential reservoirs, including cattle, companion animals, horses and pigs, and therefore conclusions regarding their origin cannot be drawn [6, 38].

Genes encoding for the production of carbapenemases were not detected, signifying that the prevalence of carbapenemase producing Enterobacteriaceae in the community is low.

## Conclusions

ESBL/pAmpC genes found in healthy humans in the community are similar to those in Dutch patients indicating that humans in the community could be a reservoir for these resistant determinants. While contact with broilers has previously been identified as a risk factor, the role of poultry in transmission to humans through the environment or the food chain remains to be elucidated.
